# Cytomegalovirus infection is associated with an increase in systolic blood pressure in older individuals

**DOI:** 10.1093/qjmed/hcw026

**Published:** 2016-04-12

**Authors:** C. Firth, R. Harrison, S. Ritchie, J. Wardlaw, C.J. Ferro, J.M. Starr, I.J. Deary, P. Moss

**Affiliations:** From the ^1^Institute of Immunology and Immunotherapy, University of Birmingham, Birmingham, B15 2TT UK; ^2^Geriatric Medicine Unit, University of Edinburgh, Edinburgh, EH16, 4SB UK; ^3^Centre for Clinical Brain Sciences, Edinburgh, UK; ^4^University Hospitals NHS Foundation Trust, Edgbaston, Birmingham, B15 2WB UK; ^5^Department of Psychology, University of Edinburgh, Edinburgh, EH8 9JZ, UK; ^6^Alzheimer Scotland Dementia Research Centre, University of Edinburgh, Edinburgh, EH8 9JZ, UK; ^7^Centre for Cognitive Ageing and Cognitive Epidemiology, University of Edinburgh, Edinburgh, UK

## Abstract

**Background:** Cytomegalovirus (CMV) is a chronic infection that is widely distributed in the population. CMV infects a range of tissues, including endothelium, and viral replication is suppressed by the host immune system. Infection is associated with increased risk of mortality from vascular disease in older people, but the mechanisms behind this have not been determined.

**Aim:** We investigated the association between CMV infection and cardiovascular phenotype in a cohort of healthy elderly donors.

**Design**: CMV serostatus and cardiovascular parameters were determined in the Lothian Birth cohort, which comprises 1091 individuals aged 70 years in whom many environmental, biochemical and radiological correlates of vascular function have been determined.

**Methods**: CMV serostatus was determined by enzyme-linked immunosorbant assay and correlated with a range of biochemical and phenotypic measures.

**Results:** Sixty-five percent of participants were CMV seropositive, which indicates chronic infection. The mean sitting systolic blood pressure (SBP) was 149.2 mmHg in CMV seropositive individuals compared with 146.2 mmHg in CMV seronegative subjects (SD 18.7 vs. 19.7; *P* < 0.017). This association between CMV infection and SBP was not attenuated after adjustment for a wide range of biological and socio-economic factors.

**Conclusions:** These data show that CMV infection is associated with an increase in SBP in individuals at age 70 years. The magnitude is comparable to environmental variables such as obesity, diabetes or high salt intake. This is the first evidence to show that a chronic infection may be an important determinant of blood pressure and could have significant implications for the future management of hypertension.

## Introduction

Cytomegalovirus (CMV) is a member of the human herpesvirus family and is widely distributed in the population.^1^ Like all herpes viruses, CMV establishes a state of chronic infection, and viral replication must be suppressed by the host immune response. CMV is a significant cause of morbidity in immunosuppressed patients but may also be associated with clinical complications in the immune competent population. CMV infection appears to carry particular significance for older adults, and infection has been associated with an increased rate of mortality in several studies.^2–5^ A particular association is with cardiovascular disease and the risk of vascular mortality has been shown to be nearly doubled in some studies.^3^ The mechanism behind this correlation is unclear although the virus can infect a wide range of cell types including endothelial and vascular smooth muscle cells.

Atherosclerosis is the major cause of vasculo-occlusive events such as myocardial infarction, but there remains debate as to whether or not CMV infection acts as a risk factor for the development of this pathology.^6^ In contrast, the virus has been associated with the development of arteriosclerosis in patients with renal disease.^7^ Arteriosclerosis is a disease of the arterial media layer in which increased collagen content, together with calcification, hyperplasia and hypertrophy of vascular smooth muscle cells, leads to arterial wall hypertrophy and increased arterial stiffness.^7^ Arteriosclerosis is characterized by an increase in systolic blood pressure (SBP). In this study, we investigated whether CMV infection was associated with an increase in blood pressure in a large healthy population at age 70 years.

## Methods

The LBC1936 study consists of 1091 relatively healthy age-homogeneous older people who participated in the Scottish Mental Survey of 1947 when aged 11 years. The participants have undergone extensive phenotypic and genetic investigation following recruitment, and the cohort has been described previously in detail.^8^ In particular, at age 70 years, participants underwent a series of cognitive tests, as well as physical and biochemical investigations. Participants were Caucasian, gave written informed consent and almost all lived independently in the Lothian region of Scotland. The data available for participants were mostly complete and where variables were missing this is stated in Table 1.

**Table 1.** Clinical and demographic characteristics of the Lothian Birth Cohort divided according to CMV serostatus

	Mean (SD)/percentages	CMV negative, mean (SD)	CMV positive, mean (SD)
CMV serostatus (%)		35.5	64.5
Gender, male (%)*	50.6	57.8	46.7
Age (years)	69.6 (0.83)	69.5 (0.88)	69.6 (0.81)
Smoking (current) (%)	13.2	15.3	12.0
BMI (kg/m^2^)	27.8 (4.35)	27.7 (4.12)	27.8 (4.43)
History of stroke (%)	5.0	5.2	4.9
Diabetes mellitus (%)	8.3	6.8	9.1
eGFR (ml/min/1.73 m^2^) *n* = 1049	82.2 (17.05)	82.3 (17.50)	82.2 (16.82)
Haemoglobin (g/l) *n* = 1049	145.2 (13.0)	146.1 (12.54)	144.7 (13.5)
HbA1c (total %) *n* = 1048	5.9 (0.77)	5.9 (0.73)	6.0 (0.79)
Triglyceride (mmol/l) *n* = 960	1.6 (0.92)	1.6 (0.80)	1.7 (0.97)
Total cholesterol (mmol/l), *n* = 1049	5.5 (1.16)	5.4 (1.19)	5.5 (1.14)
Calcium (mmol/l), *n* = 1044	2.3 (0.10)	2.3 (0.09)	2.3 (0.10)
C-reactive protein (mg/l), *n* = 1041	3.0 (4.50)	3.0 (4.50)	3.0 (4.50)

Figures are mean (standard deviation) for continuous variables, apart from C-reactive protein, which is presented as median (interquartile range) due to non-normal distribution. Categorical variables are presented as percentages, and for disease status, these represent the percentage of individuals reporting the disease. To assess differences between the CMV negative and positive groups, continuous variables were analysed by *t*-test or Mann–Whitney *U* test. Categorical variables were analysed by Chi-square. *n* = 1054 unless otherwise stated.

*P < 0.05.

Systolic and diastolic blood pressures were measured on three occasions both sitting and standing using a validated oscillometric Omron 705IT monitor.^8^ Briefly, participants were seated in a relaxed environment for up to 15 min prior to measurement and with their arm outstretched and supported. Measurements were performed in triplicate. Results are presented as the average of the second and third readings. Standing blood pressure was also assessed. All analyses were also repeated using the average of all three readings and for the individual readings.

Blood samples were taken for basic biochemistry and other measures including glycated haemoglobin (HbA1c), estimated glomerular filtration rate, thyroxine (T3, T4), albumin, cholesterol and C-reactive protein.^8^

Ankle Brachial Pressure Index (ABPI) and carotid intima thickness (intima-media thickness [IMT]) in the common carotid artery and carotid bulb and the maximum degree of any atheromatous stenosis were measured at age 73 years in a subset of individuals as previously described.^9^

### Determination of CMV seroprevalence

CMV was measured in plasma samples collected at age 70 years using a CMV enzyme-linked immunosorbant assay as previously reported.^10^ Briefly, mock and viral-infected lysate was coated onto enzyme-linked immunosorbant assay plates and plasma samples added in duplicate to plates before washing and addition of secondary antibody and substrate. Data were analysed in PRISM, and CMV titre was calculated with reference to a standard curve. Values above 10 were considered to be seropositive.

### Statistical analysis

All data were analysed using SPSS version 21.0 (SPSS Inc, Chicago, IL). Normally distributed continuous data are presented as mean and standard deviation with comparisons made using Student’s *t*-test or one-way analysis of variance. Non-normally distributed data such as C-reactive protein are presented as median (interquartile range) and analysed using Mann–Whitney *U* or the Kruskal–Wallis test. Categorical data are presented as percentage and analysed using the chi-squared test. All the variables used in the analyses had <5% of the values missing and were therefore treated as missing completely at random with case-wise deletion. Linear regression analysis was used to assess the univariable and multivariable relationship between outcomes and parameters under investigation. A type 1 error below 5% (*P* < 0.05) was considered statistically significant.

## Results

### CMV infection is present in the majority of participants

The study analysed 1091 participants of the Lothian Birth Cohort (1936), of which 37 had to be excluded due to missing CMV infection data (*n* = 30) or incomplete blood pressure measurements (*n* = 7). The demographic characteristics of the remaining 1054 study population are listed in Table 1. In total, 689 (64.5%) of the cohort were CMV seropositive and, as previously described, were more likely to be female, have lower mini-mental state scores, live in more overcrowded housing at age 11 years, be less likely to have access to indoor toilet facilities at age 11 years, their father’s occupation tended to be more manual in nature and they had fewer years of education.^11^ CMV infection was not associated with incidence of diabetes, smoking status or BMI. There were no differences in recorded haematological or biochemical variables. CMV infection did not influence the self-reported incidence of stroke or myocardial infarction.

### CMV infection is associated with an increase in SBP

**Figure 1.**
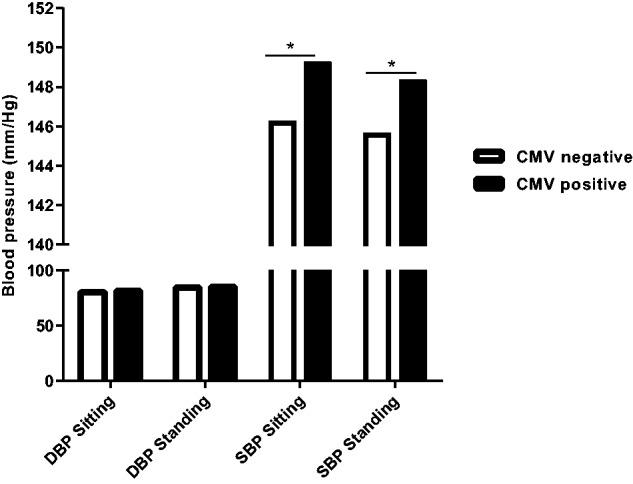
Blood pressure in relation to CMV serostatus within the study cohort. Mean diastolic pressure (DBP) and systolic pressure (SBP) were determined while sitting or standing in relation to CMV serostatus (*n* = 1054). *P ≤ 0.05.

We next went on to examine the association between CMV infection and systolic and diastolic blood pressure within study participants (Table 2). CMV seropositive subjects had a higher mean SBP and this effect was apparent when either sitting or standing. The mean systolic pressure while sitting was 149.2 mmHg in those with CMV infection compared with 146.2 mmHg in subjects without infection (SD 18.7 vs. 19.7; *P* = 0.017) (Figure 1). An increment in systolic pressure of 2.7 mmHg was observed in the standing systolic pressures, which were measured at 148.3 mmHg and 145.6 mmHg, respectively (SD 18.9 vs. 20.7; *P* = 0.032). The sitting diastolic blood pressure was over 1 mmHg higher in CMV seropositive individuals, but this value did not reach statistical significance.

**Table 2.** Cardiovascular measurements in relation to presence of CMV infection

	Mean (SD)	CMV negative, mean (SD)	CMV positive, mean (SD)
SBP (sitting)*	148.2 (19.10)	146.2 (19.71)	149.2(18.68)
SBP (standing)*	147.4 (19.58)	145.6 (20.66)	148.3 (18.93)
Diastolic blood pressure (sitting)	80.73 (10.30)	80.0 (10.36)	81.10 (10.26)
Diastolic blood pressure (standing)	84.9 (10.50)	84.3 (10.45)	85.2 (10.52)
Pulse pressure (sitting)	67.4 (15.14)	66.2 (15.04)	68.1 (15.17)
Pulse pressure (standing)	62.5 (15.48)	61.3 (15.20)	63.2 (15.60)
MAP (sitting)*	103.2 (11.88)	102.1 (12.28)	103.8 (11.63)
Pulse pressure/MAP (sitting)	0.65 (0.13)	0.65 (0.12)	0.66 (0.13)
ABPI	1.10 (0.18)	1.10 (0.18)	1.10 (0.18)
IMT (µm, right)	0.83 (0.22)	0.84 (0.24)	0.83 (0.20)
IMT (µm, left)	0.86 (0.25)	0.87 (0.26)	0.86 (0.24)
Carotid arteries maximum stenosis (%, right)	14.1 (15.65)	14.7 (15.43)	13.7 (15.79)
Carotid arteries maximum stenosis (%, left)	12.7 (14.89)	11.1 (12.74)	13.6 (15.85)

Figures are mean (standard deviation). All blood pressures are measured in mmHg. Pulse pressure (PP) was defined as SBP − diastolic blood pressure. MAP was calculated as diastolic blood pressure + 1/3 PP. Differences between CMV negative and CMV positive groups were analysed by *t*-test. *n* = 1054, with the exception of ABPIs, IMT and carotid stenosis, which were measured in 736, 798 and 506 subjects, respectively.

*P < 0.05.

Arteriosclerosis is associated with a decrease in vessel elasticity and this can be manifest as an increase in the arterial pulse pressure. We therefore examined the impact of CMV infection on pulse pressure and mean arterial pressure (MAP). CMV infection was associated with a non-significant 1.9 mmHg increase in pulse pressure, and this was observed when subjects were either sitting or standing. The MAP is the average arterial pressure during a single cardiac cycle and is calculated as the diastolic blood pressure added to one third of the pulse pressure. The MAP was increased by 1.7 mmHg in the CMV seropositive group to reach a value of 103.8 mmHg compared to 102.1 mmHg in the CMV seronegative participants (SD 11.6 vs. 12.3; *P* = 0.027).

In order to examine the potential importance of CMV infection on the development of atherosclerosis, we determined the association between infection status and both IMT and ABPI measurements. These values are widely used as indicators of atheroma load and no differences were seen in carotid IMT (*P* = 0.297), maximum carotid artery stenosis (*P* = 0.527) or ABPI (*P* = 0.401) between CMV seropositive and seronegative subjects.

### CMV infection is an independent determinant of SBP

Univariable and multivariable linear regression analyses were used to assess the relative importance of CMV infection on SBP. CMV infection was associated with SBP in univariable analysis (*B* = 2.95; 95% confidence interval [CI]: 0.53–5.37; *P* = 0.017) but was not associated with diastolic pressure (1.07; 95% CI: 0.001–0.005; *P* = 0.108). Other factors associated significantly with SBP in univariable analysis were age, gender, diabetes, haemoglobin, haematocrit, albumin, cholesterol and calcium. None of the markers of socioeconomic status that were associated with CMV infection were associated with SBP. CMV infection and the additional factors associated with SBP were then entered into a multivariable regression model. CMV infection remained positively associated with sitting SBP (*B* = 3.14; *P* = 0.011; 95% CI: 0.73–5.54).

### Assessment of CMV infection in relation to gender and medication status

Gender differences have been observed in relation to both the overall prevalence of CMV infection and the magnitude of the CMV-specific immune response within infected individuals.^1^^,^^12^ Therefore, it was important to consider the influence of gender and CMV upon blood pressure (Supplementary Table S1). Data were also available regarding whether participants were taking medication to lower blood pressure. Thirty-eight percent (262/689) of CMV seropositive individuals were taking anti-hypertensive treatment compared to 33% (120/365) of the CMV seronegative cohort (*P* = 0.098).

The increment in sitting SBP in association with CMV infection was particularly marked (6.9 mmHg) in women who were not taking anti-hypertensive medication. However, caution must be applied during analysis of subgroups, and a statistical model was therefore applied to determine if medication status or gender influenced the relationship between CMV serostatus and blood pressure. This model included CMV status, gender and medication status and both medication (*B* = 0.16, SE 0.06, *P* = 0.0125) and CMV serostatus (*B* = 0.15, SE 0.06, *P* = 0.01) were found to be associated with SBP (Supplementary Figures S1 and S2). However, there were no interactions between these three factors (*P* = 0.07–0.19) and as such the influence of CMV on blood pressure was not stronger in people taking medication or in relation to gender.

### No association is observed between CMV-specific IgG antibody titre and SBP

The titre of the CMV-specific antibody response within CMV seropositive individuals has been linked to mortality and morbidity risk in several studies.^2^^,^^4^ In our cohort, CMV-specific IgG titres were higher in female compared to male participants (1.69 vs. 1.63; *P* ≤ 0.0001). However, no significant association was seen between antibody titre and either systolic sitting (*P* = 0.16) or standing (*P* = 0.22) blood pressure. There was also no significant association when groups were split into male (*P* = 0.19) or female participants (*P* = 0.46).

## Discussion

CMV infection is associated with frailty and an increased risk of mortality in older individuals and a number of potential mechanisms have been invoked to explain this.^2^^,^^13^^,^^14^ These include immune senescence due to accumulation of large populations of virus-specific memory T cells or an increase in systemic inflammation.^15–18^ However, vascular disease is the most important pathology observed in association with CMV infection in these settings and the mechanism remains largely unknown. Our study shows that CMV infection is associated with an increase in SBP in healthy older individuals. In those patients who were not taking anti-hypertensive medication, systolic pressure was 4 mmHg higher in CMV seropositive individuals. The sitting diastolic pressure was 1 mmHg higher and this pattern would be consistent with the pathology of arteriosclerosis in which the development of vessel stiffness leads to a relative increase in pulse pressure.^19^ Such a process is likely to be sufficient to explain much of the association between CMV infection and increased risk of vascular death. Indeed, arterial stiffness is a powerful prognostic significance for cardiovascular disease as the failure to buffer intermittent left ventricular ejection into the arterial system results in left ventricular hypertrophy and fibrosis, cerebrovascular disease and renal damage.^20^

A number of biological mechanisms may be invoked to explain our observation, as the virus has a broad tropic capacity and can infect a wide variety of cells including endothelial and smooth muscle cells.^21^ Viral protein can be detected within aortic endothelial cells, and arteries may potentially serve as sites of both latent and lytic infection.^22^ Vascular pathology might occur either directly from lytic viral infection or from secondary immunopathology resulting from the immune response against infected tissue.^23^ Indeed, the majority of CMV-specific T cells are located within peripheral blood and are unique within virus-specific T cells in that they express the CX3CR1 chemokine receptor that targets them to fractalkine on endothelium.^24^ Arteriosclerosis is associated with increased arterial vessel stiffness and its clinical associations include heart failure and increased risk of vascular mortality.^25^ The pathology is also a dominant pathological feature of the vascular complications of renal disease and CMV infection is well documented risk factor for vascular disease in this setting. Indeed, recent work has identified CMV infection as a direct correlate of arteriosclerosis in this population and infection was associated with increased arterial stiffness as determined by aortic pulse wave velocity and decreased distensibility of the proximal ascending and descending aorta.^7^

An increase in murine blood pressure has been reported following murine CMV infection (MCMV) and was also found to be independent of the development of atherosclerosis. Interestingly, MCMV infection was shown to increase renin expression from the renal cells suggesting that an increase blood volume may contribute to this effect.^26^ Suggestions that CMV infection can lead to an increase in blood pressure have also been seen in human studies. Li *et al*.^27^ reported a correlation between the level of an HCMV-associated miR-UL112 and essential hypertension. In addition, a 1 mmHg increase in SBP was observed in a subgroup of men aged 24–39 years with elevated titres of CMV-specific antibody.^28^ This latter observation is intriguing as many determinants of poor health have been associated with individuals with the highest quartile of CMV-specific antibody.^2^ However, our own observations reveal a strong association between CMV infection alone and SBP, irrespective of the magnitude of the CMV-specific immune response. Moreover, we are able to define the magnitude of this effect in older adults.

An increase in systolic pressure of 3 mmHg is of marked clinical significance at a population level. An increase of only 2 mmHg is associated with a 7% increased risk of mortality from ischaemic heart disease and a 10% increased risk of mortality from stroke.^29^ Similar increments are typical of those associated with major risk factors for hypertension such as high salt intake, obesity or diabetes. The increase in blood pressure would also act as a risk factor for the development of atheroma and could explain some reports, which indicate an association between CMV infection and increased risk of ischaemic heart disease.^30^

No increase was observed in the incidence of vascular complications in relation to CMV infection in this cohort. It is likely that this reflects the relatively low prevalence of these events in the population to date. The Lothian cohort is a relatively healthy group and vascular events have proven uncommon by age 70 years. For example, 24.8% of the cohort reported a history of cardiovascular disease and 5% reported stroke. The prevalence of diabetes was 9.1% within CMV seropositive individuals compared to 6.8% in the uninfected group, but this difference was not statistically significant. Indeed, there is no evidence that CMV infection can increase the risk of development of diabetes, although the potential importance of CMV infection in established diabetes is revealed by the increased incidence of vascular complications in diabetic patients with elevated numbers of CD4 + CD28- T cells, an established correlate of CMV infection.^31^^,^^32^

CMV infection is more common in women, possibly due to an increased exposure to infants and children with recent CMV infection.^33^ Analysis of CMV-specific IgG titre in our cohort also demonstrated that titres were significantly higher in women, which may potentially reflect an increased viral load. Our data reveal a trend towards a higher SBP in CMV seropositive women, although this did not reach statistical significance. This observation should be tested in larger cohorts, but a potential association between gender, antibody titre and blood pressure would suggest that viral load or immunopathology may directly underlie the acceleration of arteriosclerosis.

Hypertension is one of the greatest challenges in modern clinical practice and, to our knowledge, our data are the clearest reported association between an infection and an increase in blood pressure. The observation that a common infection makes an important contribution to SBP offers the potential for new forms of prevention or treatment in the management of hypertension. Immunization regimens to prevent primary CMV infection are showing significant promise and offer the prospect of a vaccine to reduce the prevalence of hypertension. For those with established infection, it is even possible that anti-viral or anti-inflammatory medications may come to be valuable additions to the current lexicon of anti-hypertensive drugs.

CFi performed the CMV serotyping and contributed to statistical analysis and writing of the manuscript. RH contributed to clinical investigation of study participants. JMW provided the carotid imaging and contributed to writing of the manuscript. CFe assisted with statistical analysis and writing of the manuscript. ID and JS lead the Lothian Cohort and contributed to the design and statistical analysis of the study, as well as writing of the manuscript. PM led on study design and writing of the manuscript.

## Funding

This work was supported by Age UK, the British Geriatrics Society and the Medical Research Council.

## Supplementary material

Supplementary material is available at *QJMED* online.

*Conflict of interest*: None declared.
